# AIRR community curation and standardised representation for immunoglobulin and T cell receptor germline sets

**DOI:** 10.1016/j.immuno.2023.100025

**Published:** 2023-02-19

**Authors:** William D. Lees, Scott Christley, Ayelet Peres, Justin T. Kos, Brian Corrie, Duncan Ralph, Felix Breden, Lindsay G. Cowell, Gur Yaari, Martin Corcoran, Gunilla B. Karlsson Hedestam, Mats Ohlin, Andrew M. Collins, Corey T. Watson, Christian E. Busse, The AIRR Community

**Affiliations:** a Institute of Structural and Molecular Biology, Birkbeck College, London, England; b Human-Centered Computing and Information Science, Institute for Systems and Computer Engineering Technology and Science, Porto, Portugal; c Bioengineering Program, Faculty of Engineering, Bar-Ilan University, Ramat Gan, Israel; d Department of Biochemistry and Molecular Genetics, School of Medicine, University of Louisville, KY, USA; e Department of Biological Sciences, Simon Fraser University, Burnaby, BC, Canada; f Fred Hutchinson Cancer Research Center, Seattle, WA, USA; g Department of Microbiology, Tumor and Cell Biology, Karolinska Institutet, Stockholm, Swede; h Department of Immunotechnology and SciLifeLab, Lund University, Lund, Sweden; i School of Biotechnology and Biomolecular Sciences, University of New South Wales, Sydney, NSW, Australia; j Division of B Cell Immunology, German Cancer Research Center, Heidelberg, Germany; k Peter O’Donnell Jr. School of Public Health, UT Southwestern Medical Center, Dallas, TX, USA; l Peter O’Donnell Jr. School of Public Health, Department of Immunology, School of Biomedical Sciences, UT Southwestern Medical Center, Dallas, TX, USA

**Keywords:** Immune receptor, Immune receptor repertoire, AIRR-seq, Rep-seq, Immune receptor germline

## Abstract

Analysis of an individual’s immunoglobulin or T cell receptor gene repertoire can provide important insights into immune function. High-quality analysis of adaptive immune receptor repertoire sequencing data depends upon accurate and relatively complete germline sets, but current sets are known to be incomplete. Established processes for the review and systematic naming of receptor germline genes and alleles require specific evidence and data types, but the discovery landscape is rapidly changing. To exploit the potential of emerging data, and to provide the field with improved state-of-the-art germline sets, an intermediate approach is needed that will allow the rapid publication of consolidated sets derived from these emerging sources. These sets must use a consistent naming scheme and allow refinement and consolidation into genes as new information emerges. Name changes should be minimised, but, where changes occur, the naming history of a sequence must be traceable. Here we outline the current issues and opportunities for the curation of germline IG/TR genes and present a forward-looking data model for building out more robust germline sets that can dovetail with current established processes. We describe interoperability standards for germline sets, and an approach to transparency based on principles of findability, accessibility, interoperability, and reusability.

## 1. Introduction

**Fig. 1.**
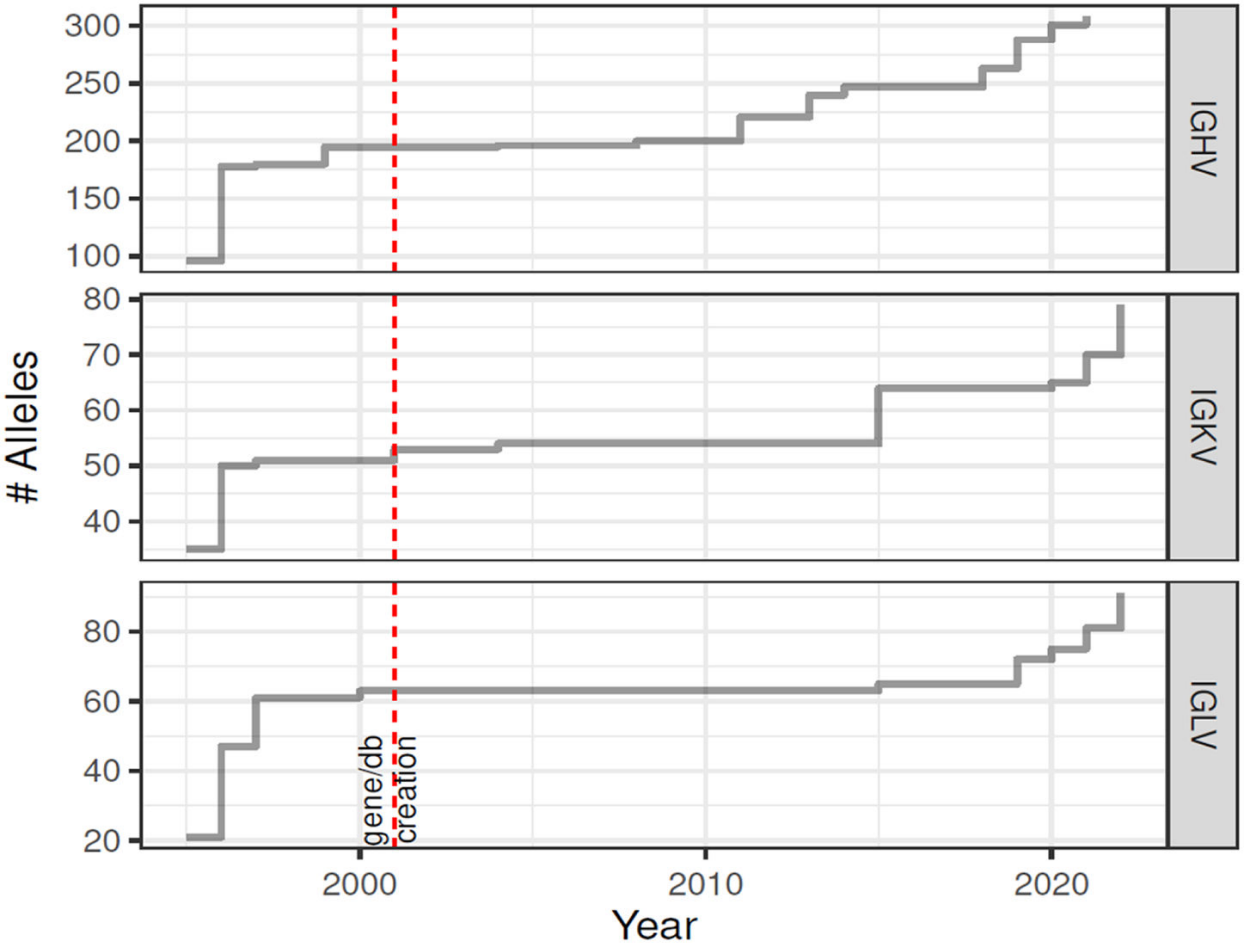
Cumulative number of human IG alleles in IMGT databases (LIGM-DB to 2001 and IMGT GENE/DB subsequently).

**Fig. 2.**
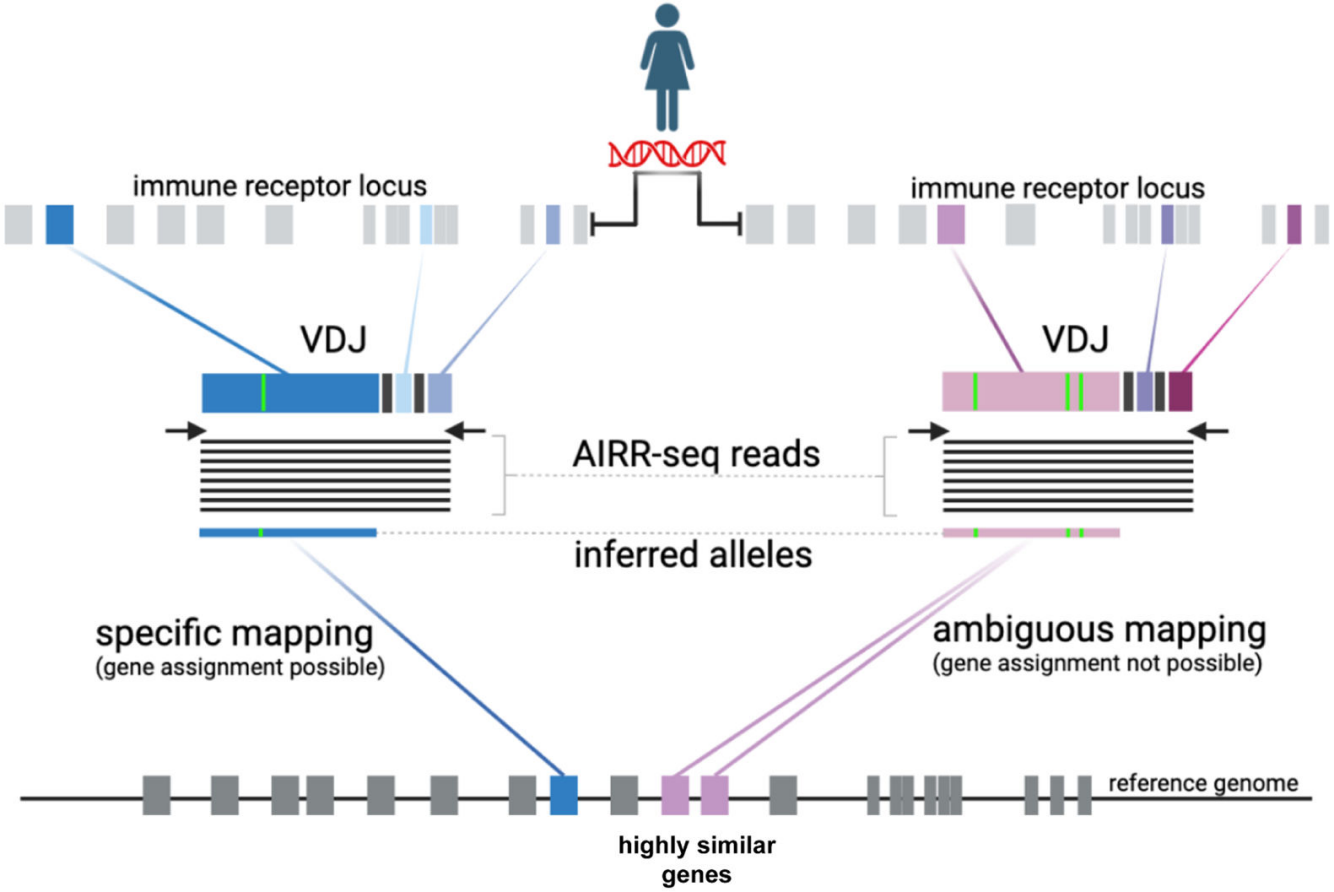
Alleles inferred from AIRR-seq reads are derived from recombined VDJ sequences, meaning that the exact genes from which they arise (determined by location in the immune receptor locus) cannot be determined. In some cases, the location can be inferred by assessing sequence similarity to genes in the reference genome. However, the presence of multiple highly similar genes may make it impossible to determine the correct mapping unambiguously.

Germline immunoglobulin and T cell receptor germline gene (IG and TR) sets are compilations of curated sequences of known germline genes and their allelic variants. These include constant (C), joining (J), diversity (D), and variable (V) genes found within the IG and TR loci of many species. Historically, germline sets have focused on sequences sourced from genomic data that meet specific criteria [1,2]. More recently, approaches have been developed that allow for the characterization of germline sequences from Adaptive Immune Receptor Repertoire sequencing (AIRR-seq) and higher throughput genomic sequencing datasets. These next-generation data sources for novel IG and TR gene and allele discovery have the potential to significantly expand existing germline sets, an important point considering that for many applications, it has been demonstrated that the use of more comprehensive germline sets can substantially improve the accuracy of data analysis, even in supposedly simple measures such as somatic hypermutation rate [3-6]. In humans, the number of IG alleles reported by the IMmunoGeneTics Information System (IMGT) [1] continues to grow each year (Fig. 1), suggesting that substantial allelic diversity remains undiscovered. Despite the advantages of combining the strengths of genomic and AIRR-seq data when deriving germline sets, the seamless integration of these data types for the construction of more comprehensive germline sets is not straightforward. This largely stems from constraints caused by current nomenclatures and curation processes, which require germline sequences to have both gene names and allele identifiers: in other words, for the sequences of alleles to be ‘mapped’ to identified genes at specific genomic locations. Germline sequences inferred from AIRR-seq, for example, cannot always be unequivocally mapped to a single location: in other words, there may be confidence that a germline sequence is observed, but not which gene it should be assigned to (Fig. 2). While fully mapped sets remain the long-term goal, there is an urgent need for mechanisms that will allow the growing body of germline sequence data discovered from next-generation sources to be published in interim form, in a codified manner that can easily be used by researchers. Such mechanisms should also allow for germline sequences and the germline sets they are part of to evolve through time transparently, as more information becomes available.

**Fig. 3.**
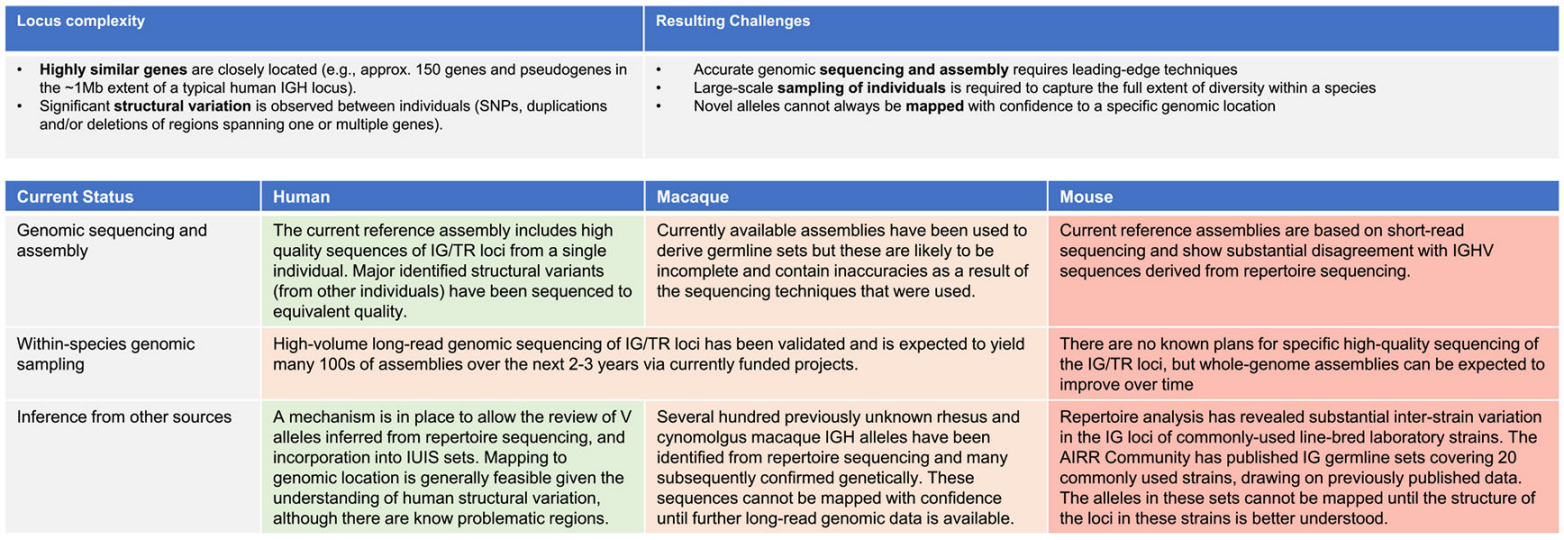
Challenges in the characterization of IG/TR genes, and the current status in three key species.

The complexity of the IG/TR loci is manifested as single-nucleotide variation in genes (allelic variation) and in non-coding regions, and, in addition, structural variation, in which whole genes or segments of the locus are deleted or duplicated [7]. The challenges are such that significant obstacles remain before complete sets can be published even for those species that have received the most attention (Fig. 3). In humans, significant structural variation is seen in the IG heavy chain (IGH) and TR beta (TRB) loci [8,9], and many structural variants have been characterised. Others are still being discovered as additional haplotypes are resolved at nucleotide resolution. Because a single reference sequence cannot represent variation in the population as a whole, the current human reference genome, GRCh38, is missing genes that are present within some IG haplotypes and hence not all recognised genes have coordinates in GRCh38. These genes are, however, represented in alternate contigs that can be properly placed relative to GRCh38 [10,11].

Compared to other species, our knowledge of the functional genes and common structural variants in the human IG loci is more complete, and thus many newly identified but unmapped sequences can be mapped following detailed review. The general principle dictates that when such a sequence aligns with known alleles of a single gene, *G*, with high sequence identity, and with substantially lower identity to the alleles of other genes, the sequence can reasonably be mapped/assigned to *G*. Such unmapped sequences can arise from traditional sequencing methods (e.g., sequencing from targeted PCR amplicons or large-insert clones), or by inference from the transcriptome. Several tools have been developed that facilitate the inference of germline alleles from AIRR-seq data [12-16]. Since their first description in 2015, these tools and the inferences produced by them have received substantial scrutiny [17-21] and there is now a consolidated understanding of the technical capabilities and limitations of the approach. Collaboration between the Inferred Allele Review Committee (IARC), of the AIRR Community (AIRR-C), the International Union of Immunological Societies (IUIS), and IMGT has allowed the incorporation of sequences inferred from AIRR-seq into IMGT’s human IG germline sets. To date, 37 inferred human IG alleles have been affirmed by the IARC, 32 of which have been included in IMGT sets.

AIRR-seq inference studies, building on earlier, foundational work based on genomic assemblies [22-24], have also enabled the discovery of large numbers of previously unknown germline allele sequences in the mouse and macaque. However, in contrast to human, our understanding of haplotype diversity in these species is limited. This knowledge gap presents challenges for curating and compiling germline sets using historical standards. We use these species here to illustrate current issues.

Commonly used inbred laboratory strains, initially derived from diverse subspecies of wild-type mice, are now understood to exhibit substantial inter-strain variation in the IG loci [5,25-27]. For example, AIRR-seq analysis has revealed that fewer than 5% of IGHV sequences curated in C57BL/6 are found in the germline repertoire of BALB/c. In the BALB/c strain, reference assemblies from the Sanger Mouse Genomes Project (https://www.sanger.ac.uk/data/mouse-genome-s-project/) were found to contain only 44% of the IGH alleles inferred by AIRR-seq [5]. Critically, these reference assemblies were compiled using short-read next-generation sequencing (NGS) [28]: the mapping and assembly of short-read data in the IG loci is problematic. This result emphasises that, even in inbred strains, the close gene spacing and repetitive nature of the IGH locus requires advanced genome sequencing and assembly techniques to identify IGH alleles reliably. While broader surveys in additional strains have identified germline alleles with the same sequence or high sequence identity in multiple strains, in the absence of genomic assemblies for these strains, it has not been possible to determine whether these alleles map to the same gene. Genomic sequencing will be required to establish gene-to-allele mappings and to establish whether a mapping to an overall structure that spans across strains is viable. At the time of writing, IG germline sets listing alleles inferred in 20 commonly used strains are available on the AIRR-C Open Germline Receptor Database (OGRDB) [29], using the new schema described in this document (Table 1).

In the macaque, Vázquez Bernat et al. [30] recently used AIRR-seq repertoires from 45 rhesus and cynomolgus macaques to uncover several hundred previously unknown allele sequences, many of which were additionally confirmed via PCR amplification of unrearranged genomic material. The results were compiled into a database, KIMDB (http://kimdb.gkhlab.se/). To investigate the potential impacts of germline databases on AIRR-seq analysis, Kaduk et al. [3] compared the annotation of a single AIRR-seq dataset annotated using the KIMDB rhesus macaque germline database with that annotated using the corresponding IMGT germline set, which is largely derived from the rheMac10 reference assembly [31,32]. Analysis with the IMGT set overestimated somatic hypermutation levels, as a result of missing genes and alleles. Further examination of the two databases demonstrated that substantial structural variation between animals was reflected in KIMDB but missing from the IMGT germline set.

In the mouse and macaque, and in other non-human species, the lack of a detailed genomic understanding means that alleles determined from transcriptomic and/or genomic information cannot as yet be mapped to genes. Their sequences serve to emphasise the current lack of understanding of the genomic structure of these two species. Hence, outside human, outlets for the efficient sharing of many identified but unlocalised germline alleles are not available, leading to confusion among researchers and impeding scientific progress and reproducibility.

While germline sets have been published for many species in addition to those covered above, they are largely based on the annotation of published reference assemblies. Experience from mouse and macaque as just summarised suggests that relying solely on a single reference assembly, especially if constructed from short-read NGS, is likely to provide poor coverage of genes and alleles, particularly in the important and complex IGH locus. Augmentation with sequences from other sources, specifically inference from AIRR-seq repertoires, can improve coverage, and also identify sequences derived from the assembly that are not observed in expressed repertoires, and may therefore be erroneous. Germline sets can therefore be improved through a hierarchy of annotation, initially starting with annotation of a reference assembly and AIRR-seq repertoires; subsequently adding further repertoires and confirming inferred allele sequences with targeted PCR amplification, cloning and Sanger sequencing, and ultimately utilising high-fidelity genomic sequencing at scale. However, this approach requires a schema that allows allele sequences to be incorporated in a germline set regardless of whether they have been mapped to a gene, in contrast to current practice.

For IG/TR loci, the close spacing of many highly similar genes and pseudogenes requires meticulous assembly and the use of longer reads than those generally employed for whole-genome sequencing (typically >8 kilobase reads, compared to the 64 or 128 base paired reads that has historically been used in short-read whole genome sequencing). Methods for high-volume, high-fidelity genomic sequencing of receptor loci are reaching maturity [33,34]. From our own work and that of others, we expect high-quality genomic sequencing of IG/TR loci from many hundreds of individuals and species to emerge over the next 2-3 years [34-36]. While alleles identified from genomic sequencing may by their nature be localised within the assembly, substantial work is still needed to integrate the results from multiple assemblies, identifying structural variants, and resolving technical errors in sequencing and assembly. Combining genomic sequencing with AIRR-seq inference of the same subjects is helpful in this respect. The combined use of advanced genomic sequencing and AIRR-seq inference currently offers the best path forward for capturing both the high population diversity in the IG/TR loci and the chromosomal location of the genes.

The schema and process outlined in this work allow unlocalised alleles to be named using a consistent naming scheme, incorporated into curated germline sets without detailed understanding of the genomic structure, and rapidly published. Allele sequences can be refined and mapped into genes as new information emerges, while preserving traceability and minimising name changes. Today, in the absence of such a framework, researchers are left to search the literature for germline sets published by particular groups, and to reconcile differences in naming and in levels of support for themselves, often discovering that the same allele has been identified in more than one study and in some cases given different names. As an example, a rhesus macaque allele identified as IGHV2-ABG*01 in [22] is listed as IGHV2-118*01 in [30].The same sequence, without the final 3’ nucleotide, was listed in IMGT as IGHV2-1*01 prior to September 2020 and is currently listed there (including the final nucleotide) as IGHV2-174*02.

In this work, we describe a schema that allows for the effective integration of genomic and inferred data without the constraint of gene mapping. It is designed to be flexible and responsive to new data types and diverse nomenclatures. By storing rich metadata alongside each sequence in the germline set, it enables traceability, cooperation between teams in the development of germline sets, and more effective integration with other genomic and immunological data types. While it is likely to require significant further development in the light of experience, it provides a foundation from which to address the large increase in data volumes and types that can be anticipated in the next few years. Finally, we define an interoperable standard format in which germline sets can be loaded into tools for gene annotation, gene usage statistics, somatic hypermutation profiling, lineage reconstruction, etc., making it easier for both users and developers to keep their tools up to date. The overall approach has been adopted by the Germline Database Working Group of the AIRR Community (AIRR-C) and is supported by AIRR-C standards and repositories as described below.

To promote transparency and re-use, we adopt the FAIR guiding principles for scientific data management [37]. To ensure that germline sets and the sequences they contain are findable, we define a schema that contains rich metadata, and assign globally unique identifiers to schema objects such as alleles and genes. For accessibility, we utilise the existing AIRR Standards-compliant infrastructure such as the AIRR Data Commons [38] and OGRDB, providing open platforms through which the germline sets can be accessed. Interoperability is currently a key problem: many tools that make use of germline sets are provided with pre-installed sets that are difficult to update: to address this, we describe a standardised format that can be used by such tools. For reusability, we include rich metadata, including fields that describe provenance, assist with traceability, and encourage open licensing. We are publishing the germline sets with an unrestricted licence and making the source code freely available.

## 2. Results

**Fig. 4.**
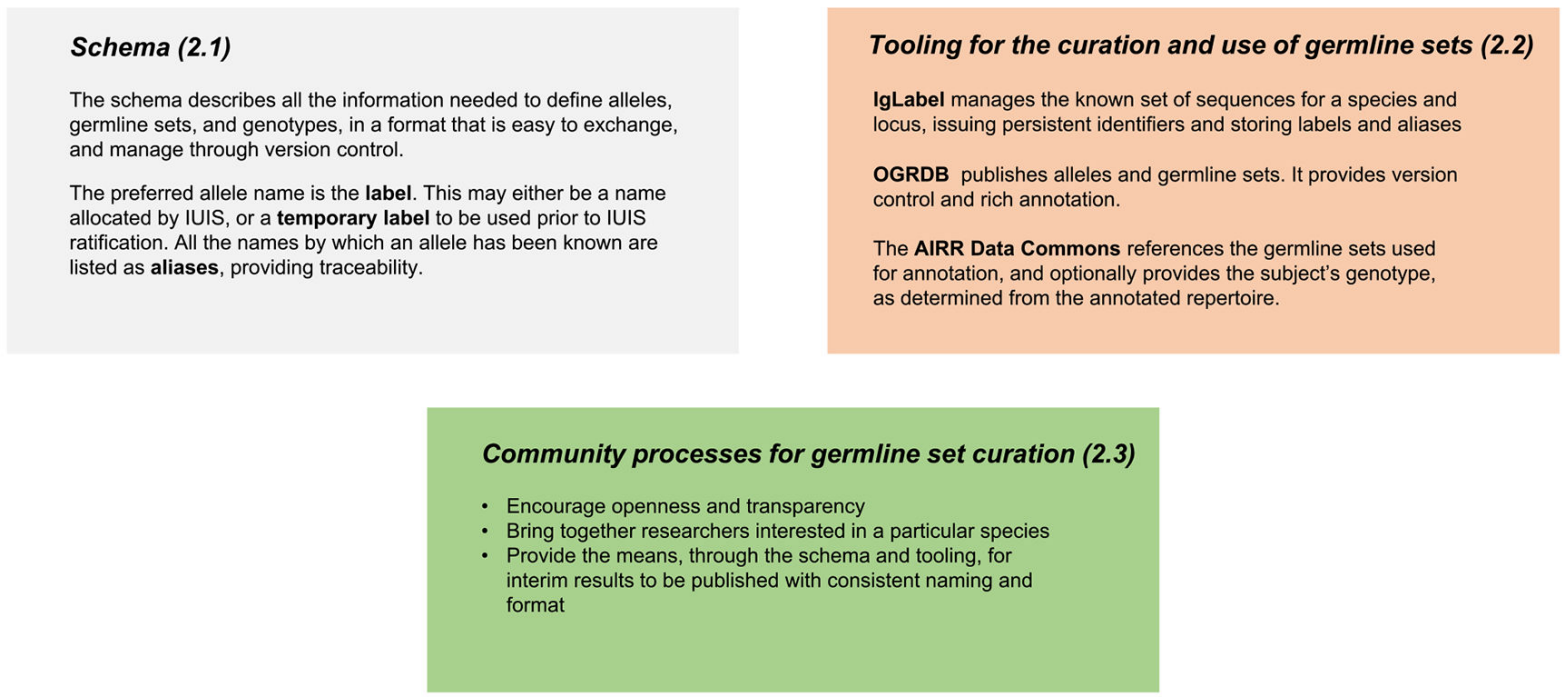
Summary of results.

The results are organised into three sections (Fig. 4):

- A schema that enables germline sets to contain rich information, and, importantly, can ensure that an identified germline sequence can be tracked through time, even if its name changes in the light of new information (Section 2.1).
- Tooling that supports germline allele review, and the publication and use of germline sets that follow the schema (Section 2 2).
- A community approach that allows researchers to co-operate in the development of germline sets in their species of interest, utilising the functionality provided by the schema and tooling (Section 2.3).

### 2.1. A schema and terminology for gene and allele curation

**Fig. 5.**
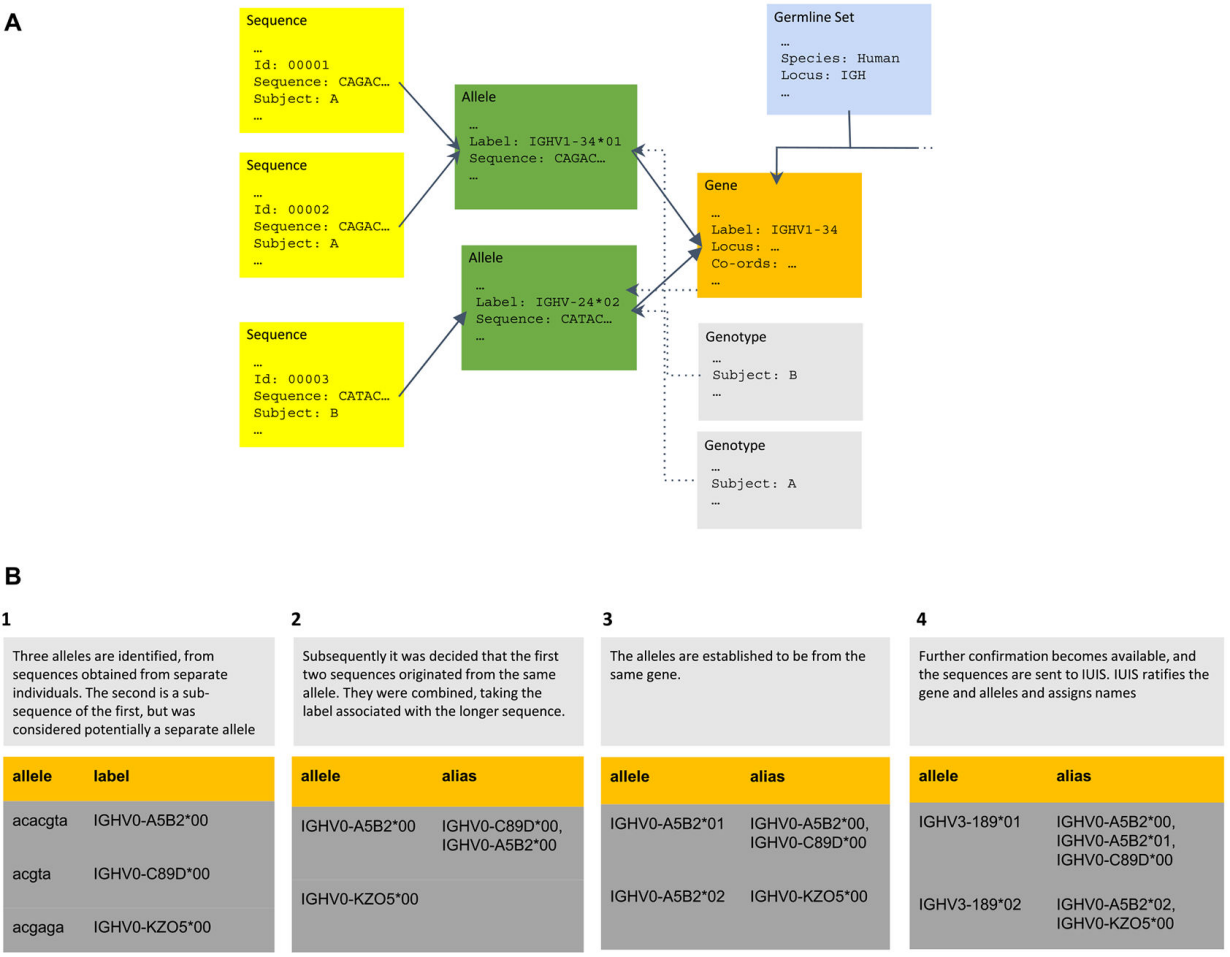
(A) – the relationship of objects defined in the Schema. (B) - evolution of labels and aliases through phases of discovery. The diagram depicts four stages in the activity of a community group curating sequences from a particular species. These events are likely to be separated in time and may be triggered by the availability of additional evidence.

As a first step towards developing the present Germline extension of the AIRR Schema, we identify common types of germline information, the names of which are used throughout the manuscript (Fig 5a):

- *Sequence:* A sequence of nucleic acids that was observed in or inferred from a single *individual*.
- *Allele*: A known – but potentially unmapped – region within the genome of at least one individual.
- *Label*: the name by which an allele is referred to in a germline set
- *Gene:* A defined and mapped region within the genome of a species that groups *alleles* based on a shared, single ancestry.
- *Genotype:* The collection of all alleles of a locus from a single *individual*, which may contain partial or complete phasing information allowing the alleles to be mapped to chromosomes.
- *Germline set:* A curated collection of genes and alleles of a single locus of a species, which may be restricted to genes and alleles of certain populations within the species.
- *Locus*: Used to distinguish the chromosomal regions in which IG/TR genes are located (e.g., IGH, TRB).

Note that in these definitions, an *individual* is a single vertebrate organism. Throughout the results, we italicise the terms above where the explicit definition is intended.

As a next step, we define the potential usage scenarios:

- Researchers can obtain current *germline sets* that support the best possible analysis of their data at that point in time.
- Researchers can refer to *genes* and *alleles* in publications using a defined nomenclature that minimises the possibility of ambiguity and supports traceability over time.
- Researchers can use germline sets to annotate AIRR-seq repertoires with the likely V, D and J *alleles* underlying each read in the repertoire, and can examine *germline sets* to understand the supporting evidence underlying each listed *allele*.
- Researchers can load *germline sets* into tools used to annotate AIRR-seq repertoires and other software at a keystroke, without manipulation.
- Software tools can produce haplotypes (i.e., fully phased genotypes) and personalised *germline sets* (sets containing just those *alleles* discovered in a single individual) in the same standard format.
- Repositories can publish the *germline sets* that were used alongside annotated repertoires, enhancing transparency and reproducibility.

#### 2.1.1. The AIRR germline set schema

**Fig. 6.**
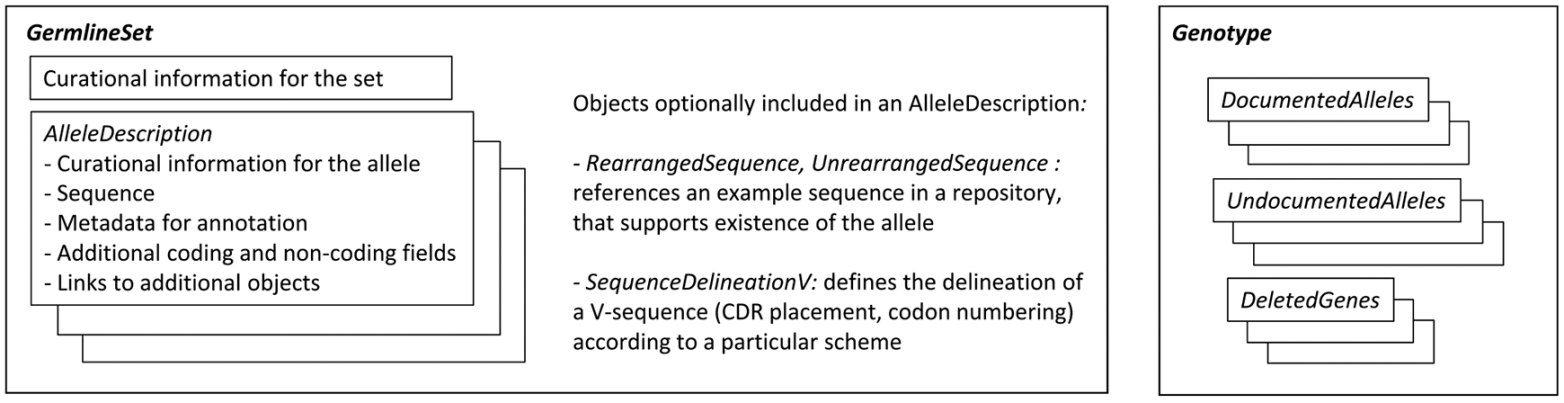
The AIRR Germline Set Schema. See Supplementary Data for detailed description and itemisation of fields.

The AIRR Data Schema [39] is maintained by open-to-all community participation via the AIRR Community Standards Working Group. The schema includes key data items associated with the processing of AIRR-seq data, defined as minimal information by the MiAIRR data standard [40]. To meet the usage scenarios, we chose to implement the above-mentioned concepts in a simplified manner that takes several specifics of gene inference workflows into account. To accomplish this, the AIRR Data Schema was expanded with the addition of two new high-level objects, *GermlineSet* and *GenotypeSet* (Fig. 6, Supplementary Information).

*GermlineSet* lists the *alleles* associated with a single *locus* of a species or species subgroup. To indicate the exact nature of genetically distinct populations within the species of interest, we included the subgroup field, which uses a controlled vocabulary (locational, breed, inbred or outbred strain) - a set of descriptors that can be extended, if necessary, to allow the curation of subgroups in all species of interest. Within the *GermlineSet*, an *AlleleDescription* is provided for each identified *allele*. Each *AlleleDescription* provides details of a single V, D, or J *allele*, describing its core sequence and, in the case of V *alleles*, IMGT alignment [41]. Fields are provided for additional coding and non-coding elements where known. The *AlleleDescription* also includes information needed for the accurate annotation of observed rearranged sequences, specifically the delineation of complementarity-determining and framework regions in V sequences using diverse schemes (e.g., Kabat, Chothia, IMGT), and the frame orientation and location of the donor splice site in J *alleles*. Additional schema objects can be associated in order to enumerate the supporting evidence for the sequence. Both *GermlineSet* and *AlleleDescription* provide fields that allow for naming, versioning and attribution.

*Genotype* describes, with reference to one or more *GermlineSets*, the specific *alleles* inferred (from repertoire analysis or genomic methods) in a single *locus* of a specific subject. Provision is made also for the identification of ‘previously undocumented’ *alleles*, that cannot be found in the referenced *GermlineSets*, and for the identification of *genes* that are not detected in the repertoire, which, depending on the analysis method and data available, may explicitly confirm that the *gene* is deleted in this individual. A ‘phasing’ field allows information to be partially or fully haplotyped, where the analysis permits.

#### 2.1.2. Temporary nomenclature

In advance of the formal recognition of a *gene* or *allele* and the approval of a name by the IUIS Reports Committee, we outline here a mechanism for assignment of standardised temporary names. We describe a concrete implementation supported by a minimal toolset, which can be evolved over time in the light of experience. In this system, when an allele is first identified, it is issued a temporary *label*. Temporary *labels* follow the IUIS style of: <locus><typexsubgroup>-<identifier>*<allele>, example: IGHV0-A5B2*00

<subroup> and <allele> take ‘null’ values of 0 and 00 respectively, until such time as specific values are determined and assigned. <identifier> is a random 20-bit value, encoded as 4-character base32 according to RFC 4648 section 6 [42]. In summary, the value is encoded as 4 characters, where each character may be an upper-case letter or a digit, but the digits 0,1,8 and 9 are omitted. This provides reasonably memorable strings, with ~1-million combinations. <identifier> is guaranteed to be unique within a naming domain and may not be re-used. The naming domain may extend to the *locus* of an entire species or may be restricted to a subgroup such as a strain or breed, at the decision of the curators. We permit the same <identifier> to be used in multiple naming domains with no overlap in meaning. For example, the same <identifier> could be assigned to an allele in humans, and also to an allele in macaques, without any implication that the two are related or share the same nucleotide sequence. Within a locus of a particular strain or species, two sequences might be identified, where one is a sub-sequence of the other (i.e., one sequence contains an identical copy of the other). In this case, the curating group can decide either to associate the same allele with the two sequences, or to associate different alleles. This might depend, for example, on whether haplotyping or usage evidence exists to support the case for the identified sequences arising from the same or different alleles. The structure of the temporary *label*, as well as being familiar to researchers, provides a level of compatibility with existing toolsets while being easily distinguishable from IUIS names.

The schema provides for *alleles* to have multiple synonyms (aliases). These are used to capture legacy designations or alternative nomenclatures of an *allele*. They are also used to store previous *labels*, where a previously published temporary *label* has been changed. Aliases therefore provide traceability across time (Fig. 5b) and may be used to integrate records across multiple germline sequence databases (e.g., OGRDB, IMGT, etc.) provided that all researchers issuing *labels* coordinate with each other, to ensure that *labels* remain unique within the naming domain. We do not propose specific rules for the process of renaming (for example, why A5B2 was preferred over C89D in step 2 of the figure), believing that it is best left to the discretion of curators.

### 2.2. Supporting tools

#### 2.2.1. IgLabel - A tool for managing the allocation of temporary labels

To support the allocation of temporary *labels* to sequences, we have developed a command-line tool, IgLabel. IgLabel uses *sequences* as input to create new or suggest existing *labels* for the corresponding *allele*, by maintaining a csv-based database for the naming domain. While originally developed to allocate *labels* to IG *alleles*, it can be used to label *alleles* in any IG or TR *locus*. New *sequences* are allocated *labels* in a two-step process. In the first step, a file listing the new *sequences* is submitted. IgLabel returns a file containing a proposed action for each *sequence*, identifying those that duplicate already-submitted *sequences*, or are sub- or super-sequences of already submitted *sequences*. In the second phase, the user reviews the actions and can optionally change them, for example to allocate a new *label* for a *sequence*, even if it is a sub-sequence of an existing *sequence*. The file of actions is then submitted and IgLabel updates the database, allocating new *labels* as needed. IgLabel is available at https://github.com/williamdlees/IgLabel under open-source licence.

It was noted previously that labels can be used to integrate records across multiple databases, provided that the issuers of labels within a naming domain coordinate with one another. This can be achieved with IgLabel if the database is published on a version control system such as Github (https://www.github.com), allowing changes from multiple sources to be merged and potential clashes handled.

#### 2.2.2. OGRDB - A system for managing and publishing germline sets

The OGRDB website was initially developed as a system to support the work of IARC in reviewing and affirming human *alleles* inferred from AIRR-seq repertoires. It has been enhanced to support the management of *sequences* identified through the review process described in this work, and their publication as *alleles*. Multiple independent review groups, such as IARC, can be supported, each with assigned responsibility for specific species and *loci*. Each group can manage tables of *sequences, alleles*, and *genes* and publish them in *germline sets*. A number of sets are already published (Table 1). The AIRR-C schema for *GermlineSet* is supported, and *germline sets* are downloadable in JSON format compliant with the schema, or in FASTA format. They are also queryable via a REST API. OGRDB manages versioning and change control, such that both users and curators can identify the addition, removal, or modification of sequences in a *germline set*, and drill down to individual records for each sequence in order to see more detail. Data published on OGRDB is provided under a minimally restrictive Creative Commons CC0 1.0 licence. OGRDB data is periodically archived at Zenodo (https://zenodo.org) for long-term storage, and each version of a *germline set* is also deposited at Zenodo and allocated a Digital Object Identifier (DOI) (ref https://www.iso.org/standard/81599.html): hence users may cite a persistent identifier that uniquely references the *germline set* used in their work. OGRDB source code is published under open-source licence.

#### 2.2.3. AIRR Data Commons

The AIRR Data Commons is a geographically distributed set of data repositories for storing and sharing AIRR-seq data that conforms to the AIRR Standards. Users can directly query and download data using the AIRR Data Commons API or using a graphical user interface such as iReceptor Gateway [43] or VDJServer Community Data Portal [44]. The AIRR schema for Subject was enhanced to allow a *GenotypeSet* to be specified for the subject, which can provide the *Genotype* for one or more *loci*. This enables repertoire queries to the AIRR Data Commons to also query any of the *Genotype* fields such as the *locus*, the *alleles*, the *germline sets* employed for annotation, and the inference process. *GenotypeSets* for one or more subjects along with their associated *Genotypes* can be downloaded in JSON format through the AIRR Data Commons API repertoire query end point.

### 2.3. A community approach to curation

With the growing interest in and usage of AIRR-seq data, *germline sets* for humans and other species are becoming widely used, but, for any species and *locus*, the number of researchers actively engaged in *germline set* curation or discovery is low. We recognise that most researchers who work with *germline sets* are invested in one or in a small number of species and wish to see those move forward, with less interest in a general approach. The schema and nomenclature outlined above provide a common and consistent framework through which researchers working with a particular species or *locus* can share and publish data in advance of formal recognition. In the absence of a group, the schema and approach can be used by an individual researcher to provide results that can be extended later.

The principles we envisage for a community approach are:

- Groups should be open to all researchers working with a particular species or *locus*. The AIRR-C Working Groups (which are open to nonmembers) provide a non-exclusive solution.
- Overlap should be discouraged, i.e., where possible, there should be just one community group working on each *locus* in a given species. In general, the small number of interested researchers should make it easy to avoid overlap: however, the schema provides approaches to nomenclature that can be used to coordinate parallel efforts where necessary, for example by storing the list of allocated *labels* in a commonly accessible and versioned repository.
- Groups should be free to determine the evidence and approach to review that best suits the overall aim of creating the best available *germline set* from the resources available, bearing in mind that the resources will vary considerably between species, and that the approach may vary considerably between inbred and outbred species or strains.
- Decision-making, supporting evidence, and review criteria should be documented and transparent. The schema provides versioning and links to records in primary repositories to support this.

## 3. Discussion

The AIRR-C is committed to the promotion of improved tools and techniques for next-generation sequencing, curation, and sharing of AIRRs [40,45]. The development and improvement of receptor germline sets is a long-standing aim in support of understanding the development of these repertoires. Eventually, the germline receptor alleles of the receptor gene loci of all species of interest may be sufficiently well characterised to the point that intermediate sets and processes such as those described here are no longer needed: this is an important long-term goal. Until it is reached, intermediate sets will provide the soundest available basis for AIRR-seq analysis, and the schema will provide transparency and traceability in results. Today, for many species, reliance on a single reference assembly for the identification of receptor alleles has yielded germline sets that do not reflect species diversity. Researchers can obtain substantially higher quality analyses by employing germline sets that more fully reflect species diversity, even if alleles are not mapped to genes. The quality of AIRR-seq analyses can further be improved using personalised germline sets provided by AIRR-seq inference tools [13,14,16], particularly when analysing highly mutated repertoires. We recommend the routine use of such tools in analysis pipelines. Some such tools, for example those that focus specifically on identifying which alleles of each gene are present in a repertoire, may need modification or extension to handle cases where the allele-to-gene mapping is not available.

Analysis tools - annotation tools, repertoire analysis tools etc. - have traditionally relied on the structure of the name to extract information such as the allele identifier, gene identifier and family designation. Indeed, the name itself has been the only source of that information in germline sets. Here we outline a schema for germline IG and TR sets which has specific fields to carry these attributes. We encourage developers to adopt the new schema, and in doing so: (1) move away from parsing information out of the name; (2) handle cases where gene identifiers and subgroup designations are not available; and (3) build tools that can easily and conveniently update germline sets by taking advantage of the new format. Germline sets are frequently updated; hence, ease of update is a factor that deserves specific attention. Likewise, strong version management is required, both in the publication of germline sets and in the attribution of results. Another factor for developers to consider is that germline sequences can be incomplete at the 5’ or 3’ end. This is a feature of current sets, and one that may persist, as AIRR-seq inferences are often inconclusive for the final nucleotides at the 3’ end. It is important, therefore, that calls are not unduly biased by sequence length [19].

Provision is made in the IMGT naming scheme for unlocalised genes via the ‘S’ gene number prefix [46], but here we describe a community process that can recognise unmapped alleles (rather than unlocalised genes) on the basis of evidence that is not currently acceptable for formal ratification. We have opted to use a naming scheme that can be easily differentiated from existing schemes. In our view, the community approach is likely to be more manageable and to lead to better results at this early stage than a more formal structure. This should not be seen to detract from the value of formal ratification: it is important that community efforts are able to support eventual ratification, and that traceability is maintained.

A consideration for curators is how comprehensive or otherwise the coverage of a germline set should be before it is published. Users can compensate for lack of completeness in gene or allele coverage by including an inference step in their annotation pipeline. Curators should provide guidance to users both on the coverage of a germline set, and on any specific considerations concerning the use of inference tools. The focus in this work has been on the documentation of previously undocumented genes and alleles, but current sets also include erroneous and incomplete allelic sequences [47]. Consideration of evidence based on AIRR-seq inference and on long-read genomic sequencing offers an opportunity for existing sets to be improved. The evidence for alleles not seen in expressed repertoires should be carefully reviewed, and partial sequences extended where evidence permits. Allele-to-gene mappings may need to be reviewed.

While the curational processes outlined here represent an important and significant step, it is only a first step. We expect the processes to change and improve over time, to be extended to C genes, which are not currently covered by the specification, and to be supported by increasingly sophisticated and interconnected systems and tools. The schema may also require modification over time to deal with complexities found in some species. The Atlantic salmon, for example, carries functional IGH loci on two chromosomes [48]. As this is thought to be a consequence of a whole genome duplication event [49], additional fields may be needed to capture the complexity of IG/TR genes in such cases.

Over time, the number of species of interest, and the volume of data available, will continue to increase. This will require novel approaches for the discovery, review, and curation of alleles from multiple data sources to build the most robust germline databases. It will need to be combined with the development of more adaptable nomenclature and data standards, as well as an accompanying schema for a distributed infrastructure that can accommodate collaborative updates from multiple sources while preserving full history and provenance.

It is important for the reproducibility and interpretation of results that germline sets used within the field are freely and openly available to all practitioners, so that reproducibility is maintained, and common standards can be established for medical and other critical applications. The underlying studies that support the development of germline sets are overwhelmingly drawn from academic research. The AIRR-C is committed to FAIR principles for data management and stewardship. Sustainability requires secure funding, however, and we call on for-profit organisations that benefit from the use of such work to develop models for funding their continued development and curation.

## Supplementary Material

1

2

## Figures and Tables

**Table 1:** Community-curated mouse germline sets currently listed on OGRDB (sets for other species will be added as available). IGHV sequences are taken from (11) and IGKV/IGLV from (23). These sets may be accessed at https://ogrdb.airr-community.org/germline_sets/Mouse.

Strain	Type	Sequences
129S1/SvImJ	IGKV	91
	IGLV	3
A/J	IGKV	102
	IGLV	3
AKR/J	IGKV	85
	IGLV	3
BALB/c	IGHV	164
BALB/c/ByJ	IGLV	3
	IGKV	98
C3H/HeJ	IGKV	96
	IGLV	3
C57BL/6	IGHV	102
C57BL/6J	IGKV	91
	IGLV	3
CAST/EiJ	IGKV	88
	IGLV	9
CBA/J	IGKV	82
	IGLV	3
DBA/1J	IGKV	104
	IGLV	3
DBA/2J	IGKV	100
	IGLV	3
LEWES/EiJ	IGKV	87
	IGLV	4
MRL/MpJ	IGKV	72
	IGLV	3
MSM/MsJ	IGKV	83
	IGLV	5
NOD/ShiLtJ	IGKV	62
	IGLV	3
NOR/LtJ	IGKV	80
	IGLV	3
NZB/BlNJ	IGKV	105
	IGLV	3
PWD/PhJ	IGKV	89
	IGLV	3
SJL/J	IGKV	67
	IGLV	3

## Data Availability

Data is publicly available at the links for software provided in the manuscript
