# Supplementary material for: AIRR community curation and standardised representation for immunoglobulin and T cell receptor germline sets
Source: Immunoinformatics (Amst). Author manuscript; Available in PMC 2023 Jun 29. (PMC10310305; doi:10.1016/j.immuno.2023.100025)
Supplement: 1 [file NIHMS1905398-supplement-1.pdf]

Germline Schema (Experimental)

Contents

- Motivation
- Receptor Germline Schema
- Gene and Allele Naming
- Genotypes
- MHC Genotypes
- File Format Specification
- GermlineSet Fields
- AlleleDescription Fields
- RearrangedSequence Fields
- UnrearrangedSequence Fields
- SequenceDelineationV Fields
- GenotypeSet Fields
- Genotype Fields
- MHCGenotypeSet Fields
- MHCGenotype Fields

Motivation

Understanding and cataloguing receptor germline genes and allele sequences is critical to the analysis of AIRR data. While the human set is relatively well understood in outline, although probably still far from complete, those of other species, even those that are relatively closely studied, is at a much earlier stage. There is an urgent need to define a standardised format for listing such genes, so that they can be shared between researchers and easily consumed by software tools.

Receptor Germline Schema

The receptor germline schema defines the data elements necessary to describe one or more receptor germline genes, together with supporting evidence. The fundamental object is the `AlleleDescription`, which describes a single gene or allele, containing the necessary details for the annotation of a rearranged sequence such as the location of CDRs (in the case of a V-gene) and framing information (in the case of a J-gene). `AlleleDescription` also contains fields to delineate RSS, and the leader regions of V-genes, should those be covered by the sequence provided.

Evidence supporting the gene or allele can be provided in linked `UnrearrangedSequence` and `RearrangedSequence` objects. Information represented in these objects will typically be stored in a repository: either an INSDC repository such as Genbank or SRA, or a lower-tier repository such as OGRDB. Please note that the key distinction between these object types is whether the V(D)J genes have rearranged, rather than the origin of the material, as mature B and T cells carry rearranged sequences in chromosomal DNA. It is most likely that supporting sequences will be `UnrearrangedSequences`, i.e. prior to rearrangement. In the case of a germline inference from a repertoire, the inferred germline sequence should be provided as a `RearrangedSequence`, if the evidence has been deposited in a repository.

For V-genes, an IMGT-gapped sequence (i.e.,. a sequence delineated in accordance with the IMGT numbering scheme) is provided in `AlleleDescription`. Other delineations, such as Chothia and Kabat, can be provided via linked `SequenceDelineationV` objects. A `GermlineSet` brings together multiple `AlleleDescriptions` from the same locus to form a curated set. The schema assumes that germline sets will be published by multiple repositories. A germline set may be uniquely referenced by means of the `germline_set_ref`, which is a composite field containing the repository id, germline set label, and version.

Gene and Allele Naming

`AlleleDescription` contains a `label` field, which should contain the accepted name for the field, as determined by the authors/curators of the record. The Nomenclature Committee of the International Union of Immunological Societies (IUIS) allocates gene symbols for receptor genes, and, if a gene symbol has been allocated, this should be used as the label. Where a gene symbol has not been allocated (for example, because the gene or allele has only recently been discovered, or because the available evidence does not meet IUIS standards, a 'temporary label' should be used. It is anticipated that publishers of gene sets will provide mechanisms to issue these temporary labels, and to allow researchers to review change history of `AlleleDescriptions` and `GermlineSets`. To provide consistency across research groups, the Germline Database Working Group of the AIRR Community is developing a community-wide approach to the allocation of temporary labels.

Genotypes

A `GenotypeSet` describes the specific receptor alleles found in a subject, and also identifies genes that are not found (this could be either because they are not present in the chromosomal locus, or because they are not expressed or expressed only at low levels). Depending on the data available and the inference method used, genotypes may contain haplotyping information, which may be full, or partial. As an example of partial haplotyping, the genotype may have been determined from genomic sequencing in which the sequence of the locus was assembled into contigs, but could not be fully assembled. In this case the co-location of alleles in each contig has been established, but the co-location across the entire locus can not be. Co-location is therefore indicated by means of the `phasing` parameter, which in this case would be assigned a different value for alleles on each contig.

MHC Genotypes

Similary to the IG/TR genotypes, the `MHCGenotype` amd `MHCGenotypeSet` objects describe the MHC alleles found in a subject. `MHCGenotype` objects assemble alleles from one class: `MHC-I`, `MHC-II` or `MHC-nonclassical`. The method used to determine the genotype can be provided in the `mhc_genotyping_method` field. As different methods might be use for the various classes, this field is located in the *MHCGenotype* object, not the `MHCGenotypeSet`.

The `mhc_genotyping_method` allows free-text descriptions, however data curators are asked to keep close to the following terms if applicable:

- `PCR-based typing`: Methods whose read-out is the amplification of specific sequences, but which do not provide sequence data by themselves. This includes SSP and SSOP.
- `Sequencing-based typing`: Clinical-grade NGS-based assays, providing high quality and resolution.
- `Inference-based typing`: Allele inferrence based on genome-wide DNA or RNA sequencing.

File Format Specification

Files are YAML/JSON with a structure defined below. Files should be encoded as UTF-8. Identifiers are case-sensitive. Files should have the extension `.yaml`, `.yml`, or `.json`.

Germline Set File Structure

The Germline Set file has a standardised structure that is utilized by all top-level AIRR Schema Objects and defined by the `DataFile` schema. It is intended to contan all information necessary to annotate receptor sequences derived from a single germline locus, and to be directly usable by annotation tools and other processing software.

The file must contain YAML or JSON representation of one or more `GermlineSet` objects, including the associated `AlleleDescription` objects. It may optionally include other associated objects: `SequenceDelineationV`, `RearrangedSequence`, `UnrearrangedSequence`, `Acknowledgement`. These should all be embedded into the overall `GermlineSet` as specified in the schema.

- The file as a whole is considered a dictionary (key/value pair) structure with the keys `Info`, `GermlineSet`, and `AlleleDescription`.
- The `GermlineSet` contains fields `release_version`, `release_description` and `release_date`, which are intended to be used for version identification, under the control of the authors of the `GermlineSet` as identified by the fields `author`, `lab_name` and `lab_address`. If the set is modified by a party other than these authors, that these 6 fields should be modified to reflect the authors of the modification, and their own version identication. These modifications MUST be made if the `GermlineSet` is, or is likely to become, public, in order to avoid confusion with the original set prior to modification. Repositories are encouraged to manage version fields automatically.
- The file can (optionally) contain an `Info` object, at the beginning of the file, based upon the `Info` schema in the OpenAPI specification. If provided, `version` in `Info` should reference the version of the AIRR schema for the file.
- The file should correspond to a list of `GermlineSet` objects, using `GermlineSet` as the key to the list.
- The file should correspond to a list of `AlleleDescription` objects, using `AlleleDescription` as the key to the list.
- There should be only one `AlleleDescription` for each allele in the list.
- Each `AlleleDescription` object should contain a top-level key/value pair for `allele_description_id` that uniquely identifies the allele description object in the file.
- Each `GermlineSet` object should contain a top-level key/value pair for `germline_set_id` that uniquely identifies the germline set object in the file.
- Some fields require the use of a particular ontology or controlled vocabulary.
- `GermlineSet` and `AlleleDescription` contain reference fields `germline_set_ref` and `allele_description_ref`. These are intended to be globally unique references (containing identifiers of the repository, object and version) that can be used in a query API.
- The structure is the same regardless of whether the data is stored in a file or retrieved from a data repository. For example, The ADC API will return a properly structured JSON object that can be saved to a file and used directly without modification.

GermlineSet Fields

Download as TSV

Name	Type	Attributes	Definition
germline_set_id	string	required	Unique identifier of the GermlineSet within this file, typically generated by the repository hosting the schema, for example from the underlying ID of the database record
author	string	required	Corresponding author
lab_name	string	required	Department of corresponding author
lab_address	string	required	Institutional address of corresponding author
acknowledgements	array of Acknowledgement	optional, nullable	List of individuals whose contribution to the germline set should be acknowledged
release_version	number	required	Version number of this record, allocated automatically
release_description	string	required	Brief descriptive notes of the reason for this release and the changes embodied
release_date	string	required	Date of this release
germline_set_name	string	required	descriptive name of this germline set
germline_set_ref	string	required	Unique identifier of the germline set and version, in standardized form (Repo:Label:Version)
pub_ids	string	optional, nullable	Publications describing the germline set
species	Ontology	required	Binomial designation of subject's species
species_subgroup	string	optional, nullable	Race, strain or other species subgroup to which this subject belongs
species_subgroup_type	string	optional, nullable	
locus	string	required	Gene locus

Name	Type	Attributes	Definition
`allele_descriptions`	array of AlleleDescription	required	list of allele_descriptions in the germline set
`curation`	string	optional, nullable	Curational notes on the GermlineSet. This can be used to give more extensive notes on the decisions taken than are provided in the release_description.

AlleleDescription Fields

Download as TSV

Name	Type	Attributes	Definition
allele_description_id	string	required	Unique identifier of this AlleleDescription within the file, typically generated by the repository hosting the schema, for example from the underlying ID of the database record
allele_description_ref	string	optional	Unique reference to the allele description, in standardized form (Repo:Label:Version)
maintainer	string	required	Maintainer of this sequence record
acknowledgements	array of Acknowledgement	optional, nullable	List of individuals whose contribution to the gene description should be acknowledged
lab_address	string	required	Institution and full address of corresponding author
release_version	integer	required	Version number of this record, updated whenever a revised version is published or released
release_date	string	required	Date of this release
release_description	string	required	Brief descriptive notes of the reason for this release and the changes embodied
label	string	optional, nullable	The accepted name for this gene or allele
sequence	string	required	nt sequence of the gene. This should cover the full length that is available, including where possible RSS, and 5’ UTR and lead-in for V-gene sequences

Name	Type	Attributes	Definition
`coding_sequence`	string	required	nucleotide sequence of the core region of the gene (V-, D-, J- or C-REGION), aligned, in the case of the V-REGION, with the IMGT numbering scheme
`aliases`	array of string	optional, nullable	Alternative names for this sequence
`locus`	string	required	Gene locus
`chromosome`	integer	optional, nullable	chromosome on which the gene is located
`sequence_type`	string	required	Sequence type (V, D, J, C)
`functional`	boolean	required	True if the gene is functional, false if it is a pseudogene
`inference_type`	string	required	Type of inference(s) from which this gene sequence was inferred
`species`	Ontology	required	Binomial designation of subject's species
`species_subgroup`	string	optional, nullable	Race, strain or other species subgroup to which this subject belongs
`species_subgroup_type`	string	optional, nullable	
`status`	string	optional, nullable	Status of record, assumed active if the field is not present
`subgroup_designation`	string	optional, nullable	Identifier of the gene subgroup or clade, as (and if) defined
`gene_designation`	string	optional, nullable	Gene number or other identifier, as (and if) defined

Name	Type	Attributes	Definition
allele_designation	string	optional, nullable	Allele number or other identifier, as (and if) defined
j_codon_frame	integer	optional, nullable	Codon position of the first nucleotide in the 'coding_sequence' field. Mandatory for J genes. Not used for V or D genes. ('1' means the sequence is in-frame, '2' means that the first bp is missing from the first codon, '3' means that the first 2 bp are missing)
gene_start	integer	optional, nullable	Co-ordinate (in the sequence field) of the first nucleotide in the coding_sequence field
gene_end	integer	optional, nullable	Co-ordinate (in the sequence field) of the last gene-coding nucleotide in the coding_sequence field
utr_5_prime_start	integer	optional, nullable	Start co-ordinate (in the sequence field) of 5 prime UTR (V-genes only)
utr_5_prime_end	integer	optional, nullable	End co-ordinate (in the sequence field) of 5 prime UTR (V-genes only)
leader_1_start	integer	optional, nullable	Start co-ordinate (in the sequence field) of L-PART1 (V-genes only)
leader_1_end	integer	optional, nullable	End co-ordinate (in the sequence field) of L-PART1 (V-genes only)

Name	Type	Attributes	Definition
`leader_2_start`	integer	optional, nullable	Start co-ordinate (in the sequence field) of L-PART2 (V-genes only)
`leader_2_end`	integer	optional, nullable	End co-ordinate (in the sequence field) of L-PART2 (V-genes only)
`v_rs_start`	integer	optional, nullable	Start co-ordinate (in the sequence field) of V recombination site (V-genes only)
`v_rs_end`	integer	optional, nullable	End co-ordinate (in the sequence field) of V recombination site (V-genes only)
`d_rs_3_prime_start`	integer	optional, nullable	Start co-ordinate (in the sequence field) of 3 prime D recombination site (D-genes only)
`d_rs_3_prime_end`	integer	optional, nullable	End co-ordinate (in the sequence field) of 3 prime D recombination site (D-genes only)
`d_rs_5_prime_start`	integer	optional, nullable	Start co-ordinate (in the sequence field) of 5 prime D recombination site (D-genes only)
`d_rs_5_prime_end`	integer	optional, nullable	End co-ordinate (in the sequence field) of 5 prime D recombination site (D-genes only)
`j_cdr3_end`	integer	optional, nullable	In the case of a J-gene, the co-ordinate (in the sequence field) of the first nucelotide of the conserved PHE or TRP (IMGT codon position 118)

Name	Type	Attributes	Definition
`j_rs_start`	integer	optional, nullable	Start co-ordinate (in the sequence field) of J recombination site (J-genes only)
`j_rs_end`	integer	optional, nullable	End co-ordinate (in the sequence field) of J recombination site (J-genes only)
`j_donor_splice`	integer	optional, nullable	Co-ordinate (in the sequence field) of the final 3' nucleotide of the J-REGION (J-genes only)
`v_gene_delineations`	array of SequenceDelineationV	optional, nullable	
`unrearranged_support`	array of UnrearrangedSequence	optional, nullable	
`rearranged_support`	array of RearrangedSequence	optional, nullable	
`paralogs`	array of string	optional, nullable	Gene symbols of any paralogs
`curation`	string	optional, nullable	Curational notes on the AlleleDescription. This can be used to give more extensive notes on the decisions taken than are provided in the release_description.
`curational_tags`	array of string	optional, nullable	Controlled-vocabulary tags applied to this description

RearrangedSequence Fields

`Download as TSV`

Name	Type	Attributes	Definition
`sequence_id`	string	required	Unique identifier of this RearrangedSequence within the file, typically generated by the repository hosting the schema, for example from the underlying ID of the database record
`sequence`	string	required	nucleotide sequence
`derivation`	string	required	The class of nucleic acid that was used as primary starting material
`observation_type`	string	required	The type of observation from which this sequence was drawn, e.g. direct sequencing, inference from repertoire
`curation`	string	optional, nullable	Curational notes on the sequence
`repository_name`	string	required	Name of the repository in which the sequence has been deposited
`repository_ref`	string	optional	Queryable id or accession number of the sequence published by the repository
`deposited_version`	string	required	Version number of the sequence within the repository
`sequence_start`	integer	optional	Start co-ordinate of the sequence detailed in this record, within the sequence deposited
`sequence_end`	integer	optional	End co-ordinate of the sequence detailed in this record, within the sequence deposited

UnrearrangedSequence Fields

[Download as TSV]

Name	Type	Attributes	Definition
`sequence_id`	string	required	unique identifier of this UnrearrangedSequence within the file
`sequence`	string	required	Sequence of interest described in this record (typically this will include gene and promoter region)
`curation`	string	optional, nullable	Curational notes on the sequence
`repository_name`	string	required	Name of the repository in which the assembly or contig is deposited
`repository_ref`	string	optional	Queryable id or accession number of the sequence published by the repository
`patch_no`	string	optional, nullable	Genome assembly patch number in which this gene was determined
`gff_seqid`	string	required, nullable	Sequence (from the assembly) of a window including the gene and preferably also the promoter region
`gff_start`	integer	required, nullable	Genomic co-ordinates of the start of the sequence of interest described in this record, in Ensemble GFF version 3
`gff_end`	integer	required, nullable	Genomic co-ordinates of the end of the sequence of interest described in this record, in Ensemble GFF version 3
`strand`	string	required, nullable	sense (+ or -)

SequenceDelineationV Fields

Download as TSV

Name	Type	Attributes	Definition
sequence_delineation_id	string	required	Unique identifier of this SequenceDelineationV within the file, typically generated by the repository hosting the schema, for example from the underlying ID of the database record
delineation_scheme	string	required	Name of the delineation scheme
fwr1_start	integer	required	FWR1 start co-ordinate in Gene Description 'alignment' field
fwr1_end	integer	required	FWR1 end co-ordinate in Gene Description 'alignment' field
cdr1_start	integer	required	CDR1 start co-ordinate in Gene Description 'alignment' field
cdr1_end	integer	required	CDR1 end co-ordinate in Gene Description 'alignment' field
fwr2_start	integer	required	FWR2 start co-ordinate in Gene Description 'alignment' field
fwr2_end	integer	required	FWR2 end co-ordinate in Gene Description 'alignment' field
cdr2_start	integer	required	CDR2 start co-ordinate in Gene Description 'alignment' field
cdr2_end	integer	required	CDR2 end co-ordinate in Gene Description 'alignment' field
fwr3_start	integer	required	FWR3 start co-ordinate in Gene Description 'alignment' field
fwr3_end	integer	required	FWR3 end co-ordinate in Gene Description 'alignment' field
cdr3_start	integer	required	CDR3 start co-ordinate in Gene Description 'alignment' field
alignment	array of string	optional, nullable	one string for each codon in the fields v_start to cdr3_start indicating the label of that codon according to the numbering of the delineation scheme

By AIRR Community
© Copyright 2017-2021, AIRR Community.

GenotypeSet Fields

[Download as TSV]

Name	Type	Attributes	Definition
receptor_genotype_set_id	string	required	A unique identifier for this Receptor Genotype Set, typically generated by the repository hosting the schema, for example from the underlying ID of the database record
genotype_class_list	array of Genotype	optional, nullable	List of Genotypes included in this Receptor Genotype Set.

Genotype Fields

Download as TSV

Name	Type	Attributes	Definition
receptor_genotype_id	string	required	A unique identifier within the file for this Receptor Genotype, typically generated by the repository hosting the schema, for example from the underlying ID of the database record
locus	string	required	
documented_alleles	array of object	optional, nullable	Array of alleles inferred to be present which are documented in GermlineSets
undocumented_alleles	array of object	optional, nullable	Array of alleles inferred to be present and not documented in an identified GermlineSet
deleted_genes	array of object	optional, nullable	Array of genes identified as being deleted in this genotype
inference_process	string	optional, nullable	Information on how the genotype was acquired. Controlled vocabulary.

MHCGenotypeSet Fields

Download as TSV

Name	Type	Attributes	Definition
mhc_genotype_set_id	string	required	A unique identifier for this MHCGenotypeSet
mhc_genotype_list	array of MHCGenotype	required	List of MHCGenotypes included in this set

MHCGenotype Fields

Download as TSV

Name	Type	Attributes	Definition
mhc_genotype_id	string	required	A unique identifier for this MHCGenotype, assumed to be unique in the context of the study
mhc_class	string	required	Class of MHC alleles described by the MHCGenotype
mhc_alleles	array of object	required	List of MHC alleles of the indicated mhc_class identified in an individual
mhc_genotyping_method	string	optional, nullable	Information on how the genotype was determined. The content of this field should come from a list of recommended terms provided in the AIRR Schema documentation.
