# Supplementary material for: AIRR community curation and standardised representation for immunoglobulin and T cell receptor germline sets
Source: Immunoinformatics (Amst). Author manuscript; Available in PMC 2023 Jun 29. (PMC10310305; doi:10.1016/j.immuno.2023.100025)
Supplement: 2 [file NIHMS1905398-supplement-2.docx]

| **Name** | **Affiliation** |
| --- | --- |
| Open-Ended Response | Open-Ended Response |
| Justin Barton | Institute of Structural and Molecular Biology, University of London |
| Eric Waltari | CZ Biohub |
| Jahn Zhong |  |
| George Blanck | USF |
| Steven Kleinstein | Yale School of Medicine |
| Andrew Farmer | Takara Bio USA, Inc. |
| Aaron Rosenfeld | University of Pennsylvania |
| Nina Senna | Omniscope |
| Khang Lê Quý | University of Oslo |
| Justin T. Kos |  |
| Swati saha | University of Louisville |
| Melissa L. Smith | University of Louisville |
| Uddalok Jana | University of Louisville |
| William Gibson | University of Louisville |
| Eve Richardson | University of Oxford |
| Puneet Rawat | University of Oslo |
| Bojan Zimonja | SFU |
| Kira Neller | SFU/iReceptor |
| Ida Lindeman | Oslo University Hospital |
| Sebastiaan Valkiers | University of Antwerp |
| Ayelet Peres | Bioengineering Program, Faculty of Engineering, Bar-Ilan University, Ramat Gan, Israel |
| Gur Yaari |  |
| Noah Yann Lee | Yale University |
| Jinwoo Leem | Alchemab Therapeutics |
| Jake Galson | Alchemab Therapeutics Ltd |
| Justin Barton | Alchemab Therapeutics |
| Kenneth Hoehn | Yale School of Medicine |
| Ademar Aguiar | University of Porto |
| Artur Rocha | INESC TEC |
| Dylan Duchen | Yale University |
| Hailong Meng |  |
| Cole Jensen | Yale University |
| Edward Lee | Yale University |
| Edel Aron | Yale University |
| Azahara Fuentes | PhD student INCLIVA (valencia, Spain) |
| Habib Bashour | the University of Oslo |
| Simon Schaefer | FAU Erlangen |
| Rahmad Akbar | University of Oslo |
| Ghadi Al Hajj | University of Oslo |
| Andrei Slabodkin | University of Oslo |
| Maria Chernigovskaya | UiO |
| Imran Fanaswala | University of Zurich |
| Pieter Meysman | University of Antwerp |
| Nicky de Vrij | UAntwerp |
| Mats Ohlin | Lund University |
| Akhila MELARKODE VATTEKATTE | University of Paris Cité and University of Réunion Island |
| Jason Vander Heiden | Genentech, Inc |
| Nina Luning Prak | Perelman School of Medicine, University of Pennsylvania |
| Brian Corrie | Simon Fraser University |
| Pei-Lung Chen | Graduate Institute of Medical Genomics and Proteomics, National Taiwan University |
| Katherine Jackson | Garvan Institute of Medical Research |
| Rodrigo García-Valiente | Amsterdam UMC location University of Amsterdam, Epidemiology and Data Science, Meibergdreef 9, Amsterdam, Netherlands |
| Lonneke Scheffer | University of Oslo |
| Ivana Mikocziova | Turku Bioscience Centre, University of Turku and Åbo Akademi University, FI-20520 Turku, Finland |
| Sheena N. Smith | Vector BioPharma AG |
| Shiyu Wang |  |
| Savita Jayaram | MedGenome Labs |
| Johannes Trück | University Children's Hospital and Children's Research Center, University of Zurich, Switzerland |
| Mariotti-Ferrandiz | Sorbonne Université |
| Cédric R. Weber | Alloy Therapeutics |
| Philippe A. Robert | University of Oslo |
| Eric de Sousa | Champalimaud Foundation |
| Anne Eugster | CRTD TU Dresden |
| Ulrik Stervbo | Marien Hospital Herne – Universitätsklinikum der Ruhr-Universität Bochum |
| Theam Soon Lim | Institute for Research in Molecular Medicine, Universiti Sains Malaysia, 11800 Malaysia |
| Wei Zhang | City university of Hong Kong |
| Chaim A Schramm | Vaccine Research Center, NIAID, NIH |
| Brian G. Pierce | University of Maryland |
| Scott Christley | UT Southwestern Medical Center |
| Jamie Scott, MD, PhD | Simon Fraser University (emerita prof) |
| Gur Yaari | Bar Ilan University |
| James Heather | MGH |
| Daniel C. Douek | National Institutes of Health |
| Victor Greiff | University of Oslo |
